# Community engagement in biomedical research in an African setting: the Kintampo Health Research Centre experience

**DOI:** 10.1186/1472-6963-13-383

**Published:** 2013-10-03

**Authors:** Kwaku Poku Asante, Charlotte Tawiah Agyemang, Charles Zandoh, Jacob Saah, Lawrence Gyabaa Febir, Casimir Kabio Donlebo, Seth Owusu-Agyei

**Affiliations:** 1 Kintampo Health Research Centre, Ghana Health Service, P. O. Box 200, Kintampo, Brong Ahafo Region, Ghana

**Keywords:** Community, Engagement, Health, Research, Ghana

## Abstract

**Background:**

Community engagement (CE) is becoming relevant in health research activities; however, models for CE in health research are limited in developing countries. The Kintampo Health Research Centre (KHRC) conducts research to influence health policy locally and also internationally. Since its establishment in 1994 with the mandate of conducting relevant public health studies in the middle part of Ghana, KHRC has embarked on a series of clinical and operational studies involving community members. In these studies, community members have been engaged through community durbars before, during and also after all study implementations. Lessons learnt from these activities suggested the need to embark on further CE processes that could serve as a model for emerging research institutions based in African communities.

**Methods:**

Interactive community durbars, workshops, in-depth discussions, focus group discussions and radio interactions were used as the main methods in the CE process.

**Results:**

Community members outlined areas of research that they perceived as being of interest to them. Though community members expressed continual interest in our traditional areas of research in communicable, maternal, neonatal and child health, they were interested in new areas such as non- communicable diseases such as diabetes and hypertension. Misconceptions about KHRC and its research activities were identified and clarified. This research provided KHRC the opportunity to improve communication guidelines with the community and these are being used in engaging the community at various stages of our research, thus improving on the design and implementation of research.

**Conclusion:**

KHRC has developed a culturally appropriate CE model based on mutual understanding with community members. The experience obtained in the CE process has contributed to building CE capacity in KHRC. Other health research institutions in developing countries could consider the experiences gained.

## Background

Community Engagement (CE) in research has been interpreted in various ways [1-3]. Basically, it is a process of involving populations in a defined area in research; identifying priority interventions within a social context and the environmental problems as well as implementing intended interventions in a culturally acceptable manner. CE needs to be an integral part of all public health research as it helps to bridge the gap in the community’s understanding, expectations and perceived needs of the research on one hand, and the scientist’s understanding of the community’s research needs in a given socio-cultural context [4]. CE process may also promote an early uptake of research results by the community to help improve health. Though other factors such as poor research design may jeopardize health research, poor CE processes may also lead to premature termination of health research or programmes as occurred during clinical trials in Cambodia and Cameroun [5,6]; mass de-worming programme in Ghana [7], and polio eradication programmes in northern Nigeria [8]. Pragmatic models for implementing CE process are required in health research and there is the need to document practical efforts especially in resource poor settings such as rural Ghana.

The Kintampo Health Research Centre (KHRC) is a Ghana Health Service research institution located in the middle part of Ghana. Its mandate is to conduct public health research aimed at influencing health policy in Ghana and internationally. Since its establishment in 1994, the KHRC has been conducting most of its research in a largely rural area in the middle part of Ghana; focusing on studies in maternal, neonatal and child health, mental health and health care financing [9-24]. CE activities have been core to KHRC’s research activities and are always carried out prior to, during and following each research project as and when research grants are obtained. However, our approach in the past had been project specific and not about KHRC’s research agenda in total. For example, in a maternal mortality study, community members were approached to discuss study related issues such as study implementation plan. We report on our new approach in engaging community members in defining our research agenda as part of KHRC’s five-year strategic research plan and discuss the CE process. We also sought community’s perceptions and expectations of the KHRC’s research activities. We believe our report will serve as a guide to CE in other research institutions in developing countries.

## Methods

### Description of KHRC study population

The core research area of KHRC comprise of two districts, Kintampo North Municipality and Kintampo South District which cover a total area of 7162 km^2^ with a resident population of approximately 142,000 based on the Kintampo Health and Demographic Surveillance Systems [25]. The area is located in the middle belt of Ghana where community members are predominantly subsistent farmers. About 70% of the population live in rural areas which are very diverse in terms of ethnicity with the two main ethnic groups being Bonos and Mos. Less than half of household heads have undergone formal education [26].

### Objective of the CE process

The objective of our CE activity was primarily to involve community members in defining a research agenda based on diseases that affect them. The outcome of the study was included in KHRCs research agenda and KHRC’s 5 – year strategic plan. In the process we also sought to identify the communities awareness of KHRC, their perceptions and expectations of KHRCs research agenda.

### Community members and stakeholders in the CE process

The target population for the CE process was mainly defined as community members in KHRCs study area that could potentially be involved in research as participants and their key gatekeepers such as community committee members, local government representatives and political leaders known as assembly members, the media, health workers and other government agencies in both the Kintampo North and South District Assemblies [27].

### CE activities

#### Preparatory phase

A project team comprising of KHRC’s research scientists, communication experts and members of management was formed. This team was responsible for the project implementation, monitoring and evaluation. An implementation framework was developed by the project team with emphasis on time schedules and responsibilities that guided the team to a successful achievement of the objective of the project. Communication tools such as message leaflets were developed to describe KHRC’s research agenda, ongoing studies and contact details of KHRC’s communication unit. The leaflets were pre-tested and distributed during the CE process. A 15 minute documentary was prepared to demonstrate KHRC’s research agenda, facilities and study implementation procedures. For example, the documentary demonstrated blood sample collection and processing at the KHRC laboratory. A drama was produced in the local language using local characters to further demonstrate KHRC’s key activities. The drama served as a source of attraction and initiation of discussions during community durbars. Ethical approval was obtained from KHRC’s Institutional Ethics Committee and the Ghana Health Service Ethical Review Committee prior to the implementation phase.

#### Implementation phase (CE methods)

Various methods were used in the CE process that appropriately targeted various members of the community. This included focus group discussions (FGDs), one on one discussions with community opinion leaders, radio discussions, community durbars and workshops. All discussions were tape recorded and transcribed by research officers. Twenty-four focus group discussions and 29 individual discussions with opinion leaders were conducted between February 2009 and October 2009 (Table 1). Participants were purposively selected to represent varied ethnicities, communities and participation in the KHRC research activities.

**Table 1:** Target populations and approach used in reaching target population

**Target population**	**Approach**	**Number conducted**	**Target population reached**
Male and female adolescents	FGD	6	48
Women with children under 5 years	FGD	6	32
Men whose children have or have not been involved in any study conducted by KHRC	FGD	6	37
Women 45 years+	FGD	6	43
Traditional leaders	IDI	2	2
Assembly men and women and government appointees.	IDI	12	12
Head of district commission for human rights and administrative justice.	IDI	1	1
District Information officer.	IDI	1	1
Church leaders	IDI	2	2
Ghana Private Road Transport Union (GPRTU ) Official	IDI	1	1
Health professionals	IDI	3	3
Traditional healer	IDI	1	1
Director of Agriculture	IDI	1	1
Unit committee members.	IDI	1	1
Chemical drug sellers	IDI	2	2
Ghana Police personnel	IDI	2	2
Community members	Radio discussions	7	~52,000
Community members	Community durbars	33	~86,700
Assembly members, the media, health professionals and other government organizations	Workshops	5	145

#### Radio discussions

Radio discussions were held in the evenings when household members were back from their daily economic activities in the two major languages spoken in the study area. Community members were welcome to phone into the programme to ask questions or contribute to the discussions. The research team also sought for the community’s feedback on past and ongoing studies.

#### Community durbars

A community durbar is a gathering of community members with their leaders to celebrate an occasion or hold a general meeting. It is usually organised by the community opinion leaders (Chiefs, Queen mothers, Assemblymen among others) to discuss issues concerning the community and also to disseminate information about the community. The research team arranged with community leaders to organise community durbars in about 33 clusters of communities. On the day of a durbar, a KHRC documentary was shown to the community members. A research officer moderated discussions with community members of their opinions, perceptions and expectations of the KHRC’s research activities. Women were especially encouraged to talk at these durbars because traditionally women do not talk in the midst of their husbands or men in general.

### Workshops

A day’s workshop each was held for the District Assemblies, District Health Professionals in Kintampo North and South districts; the local media and other community based departments (Agriculture, Education, Security, and Human Rights).

#### Analysis

Qualitative data recorded during FGDs, one on one discussions with community opinion leaders, radio discussions, community durbars and workshops were grouped and categorised using QRS NVIVO version 7 qualitative software. Responses were analysed to identify themes that addressed the study’s objectives and emerging themes for each of the qualitative methodologies used. Quotes that best describe the various themes identified are included in the results.

## Results

### Awareness of KHRC

Community members and other stakeholders associated with the various aspects of research activities carried out or being carried out in their communities were aware of KHRC was also perceived as an institution that helps to improve the health of community members. The research mandate of the KHRC was well known among government officials who were interviewed. One of the officials who had been involved in one of the research activities of KHRC had this to say:

“I have heard a lot about KHRC and I know they carry out research activities. I am aware of a research on mental health which they have just started and got invited as one of the stake holders” (IDI, District Representative)

KHRC was associated with provision of health care for women and children. Women in areas where malaria drug and vaccine studies were conducted associated KHRC to the free health care provided as part of the study procedures as indicated below:

“In this community we are very interested in the malaria project because our children are treated for free when they are sick and also they are provided free transport to the health facility and the food that is given when they are sick” (FGD, Woman with a child less than 5 years of age).

KHRC was associated with other specific studies of long duration such as the Kintampo Health Demographic Health Surveillance System that started in 2004 and was also referred to as *“Obaapa”*. *“Obaapa”* was the name of a study that was conducted in the study area for 8 years to determine the effects of vitamin A supplementation on maternal mortality among women in the fertile age group. The activities of these trials were used as an identity for KHRC.

“Sometimes too, I see them going round town counting people and issuing yellow cards to people. They use to come back every year to do the counting again and again…They also come here sometimes to ask us questions about our names, ages and the number of children that we have” (53 year old male, FGD)

“Yes, I have heard of KHRC and I have been invited for their workshop in which a forum was organized for pregnant women. I know they carry out Obaapa vita program” (Community Chief, Rural Area).

KHRC was also associated with incentives that participants receive as part of their participation in trials. For instance, packed lunch is provided to participants when they attend clinic visits for specific trials.

“They give people incentives such us ‘take away’ [packed lunch] during their interviews they inquire about whether we or our children fell sick over the last month when they visited us” (FGD, woman with a child <5 years).

About 51,900 community members had the opportunity to watch KHRC’s video documentary her on the research activities.

Awareness of the KHRC’s research activities was enhanced after the video shows. Community members emphasized the relevance of the video show and encouraged KHRC to organise similar activities on regular basis.

“I encourage the authorities of the KHRC and the organisers of this programme to continue to organise such programmes as they tend to inform the community members about the research activities of KHRC” (a female community member)

### Issues identified during CE process on KHRC research agenda

Enhancing diversity: It was identified that KHRC’s current research activities are currently concentrated on women and children. Community members suggested an expansion of the current research agenda to include all age groups and men as indicated by a participant below.

“There is too much concentration on women and children to the neglect of the health of the men. We also want the Centre to begin to do research on men because they also have health problems. The research normally does not involve all age groups and so there is the need to involve all age groups of women (adult male, FGD)”.

“Our husbands were not all that happy that they were not given Obaapa Vita [Vitamin A/ placebo capsules]. So I wish you give them something to boost their “power” instead of concentrating on only women and children because they are responsible for making us pregnant” (FGD, Woman with a child less 5 years old’).

Other diseases such as *Kwashiokor*, cholera, piles, HIV/AIDs, stroke, Onchocerciasis and maternal deaths were mentioned as other priority research areas. These diseases were identified because community members mentioned them as common in the communities or severe when they occur. Determinants of health such as environmental factors associated with diseases such as water sources were also mentioned as important areas to be researched.

“Our water source is highly polluted because the livestock graze around the dam and drink from the same source as the human beings. Please, do some studies on this to educate us on how to take good care of the dam so we don’t get diseases” (FGD, Adult males)

KHRC’s research activities were considered to address the health needs of the communities such as provision of free health care to study participants and community health education. Most discussions however identified the need to do studies that will also bring along resources such as provision of new community clinics and jobs for the youth within the communities.

### Strengthening CE

The methods of engagement between KHRC, the community and other stakeholders were discussed during the process. Participants were generally happy for the opportunity to discuss and contribute to KHRC’s research activities. They suggested regular distribution of news letters to district assembly members or government departmental heads and a stronger collaboration with the local information service department as ways to enhance engagement with all stakeholders in the study area.

“I commend you for taking the initiative to undertake this project (CE project) which has brought us together to share ideas and to listen to and learn the activities of KHRC. I am particularly happy about the leaflets which contain the research activities and agenda of the centre and the film show which summarises the research activities and agenda of KHRC”. (Government appointee, Kintampo North Municipality)

“There should be a strong collaboration between KHRC and the local Information Services Department. The communities are sometimes not cooperative to individuals because they do not know who is coming but if they see the ISD van, they will know that a government agency is coming and they will listen with all ears. You should also use the ISD to disseminate information to the general public”. (Retired educationist, Kintampo South District).

Potential challenges to KHRC research agenda: There were however potential challenges identified that may influence the implementation of KHRCs research agenda. These were identified from community members’ experience as participants in existing or previous studies. Community members indicated that blood samples collected as part of biomedical research was perceived to be sold to foreigners as narrated below:

“The blood samples that are taken from the children are sent overseas to be given to the older people so that they will become strong and healthy. This is because, it is only women who are below 45 years who are involved in the study [Obaapa Trial, Maternal mortality trial] so their blood is already strong, and so after investigating the blood samples you send them abroad to be given to the older people to make them feel healthy. They say the blood of the babies is compatible with that of the older people abroad who also need improvement in their health to live longer”. (FGD Men)

Some questions relating to personal assets were thought to create some expectations which are never met by researchers. Additionally, some questions were thought to be personal and embarrassing to respondents. This includes questions on marital status among adolescents. This unmet expectations and personal questions were thought to affect participation in future studies planned in KHRC’s research agenda if not ascertained in a culturally acceptable manner.

“We would like it if the questions about our parents assets are taken off. Sometimes when they come to ask these questions, the women think that the centre will give them those items but they would wait in vain” (FGD, Male adolescent)

“I have a friend who complained that she did not like the question that explores whether or not she is married and whether the customary rites have been performed. She said because of these questions she did not want to be part of any study. I would like that aspect to be taken off”. (FGD, female adolescent)

Disappointments of not being included in studies: Community members were said to be disappointed if they were not included as participants in previous studies.

Some of your studies are done in a small area and it will be better if you extend the area. I was once told by one of your workers that a study is going to be carried out but the computer has already selected some names but not others. Some people were not happy and complained a lot. (IDI, community assembly member).

### KHRC experiences in CE

Prior to embarking on this CE activities, KHRC’s research agenda and activities were discussed mainly with other health officials and less often with community members. This programme provided a great opportunity to comprehensively discuss KHRC’s research agenda/activities and seek the communities’ opinions on them. The capacity for CE is enhanced within KHRC. Suggestions made by community members and other stakeholders have been included in KHRC’s five year strategic plan.

Use of communication tools such as powerpoint presentations were not useful in communicating with community opinion leaders during workshops. Powerpoint presentations were to be didactic and scientific. The workshops were therefore organised informally and this aided discussions and brought several themes that are beneficial to KHRC.

From our interactions with community members, about 80% of community members get their information from both radio discussions and community durbars [28]. The two methods have been used by KHRC and the District Health Administration in public health education. From the experience in the KHRC’s study population, radio phone–in discussions is a good method of engaging community members in large communities where community durbars are difficult and expensive to organise. On the other hand, members of some communities can only listen to radio discussions but are unable to call in due to lack of telephone coverage. In these areas, community durbars are an appropriate engagement tool.

Socio-cultural norms of the communities are essential in the CE process. In the KHRC study area, it is uncommon for adolescents to discuss issues with adults. Separate FGDs were therefore organised for adolescents, women and men to enhance peer discussions and gain the best responses. Women and adolescents were however encouraged to contribute to discussions after the video shows. Traditionally, there are differences in gender representations at community durbars. Women were expected to be under represented and less vocal. Contrary to our expectations, a significant proportion of women attended the community durbars and made contributions about their experiences with KHRC activities.

### Key challenges

Unlike qualitative and quantitative research that has clear guidelines for ethical review, the nature of CE was more of a discussion than a research study. However, during our discussions with the ethics committee, it was suggested that there was the need to seek ethical approval because 1) the project required interacting with community members, and 2) information gathered will be summarized and shared with other researchers and the scientific community outside the project team. The proposal was subsequently submitted to the KHRC Scientific and Ethics Review Committees for approval prior to the start of study activities.

It was observed that community members found it difficult to differentiate between various studies by just mentioning the study names and without a brief description of some specific study procedures. This may be as a result of challenges in adequately translating medical terminologies into local languages by the study team during the CE process. Additionally, there was a challenge in distinguishing research activities that included health care from routine health service delivery. For instance participants in a clinical trial associated clinical care received as part of the research as provision of free health service.

Access to the communities was a challenge. Majority of the communities have very poor road network which that got worse during the rainy season. The only way this could be overcome is by the use of a 4x4 cross-country vehicle that could withstand trenches and holes in the roads or scheduling work during dry periods when the road network was motorable.

The audiovisuals used for the video shows in the evenings required an electricity source. Some communities did not have electricity and in places where electricity was available, there were power outages during the film shows. This challenge was overcome by always using a mobile generator as the main power source or a back-up.

## Discussion

We discussed KHRCs intended research agenda to be included in her 5 year strategic plan. The process was different from previous community engagement processes that were conducted prior to specific research studies and dwelled on the specific study processes. In this current process our aim was to involve community members and stakeholders in deciding on KHRC’s research agenda. In the process we sought the awareness and perceptions of the communities about KHRC. This approach is the first of its kind being used in KHRC. The inclusion of community members in the process of defining research agenda for research centres will make the research being conducted relevant to them as has been demonstrated in Kenya [29] and South Africa [30].

Discussions on the community perception of KHRC dominated the CE process as compared to discussions on KHRC’s research agenda probably because the process provided a first unique opportunity for community members to feedback to KHRC. In our study area, we received a positive opinion from community members regarding past studies that are conducted in the study area. It is likely that, these opinions are biased due to challenges among community members in distinguishing between clinical care received through research activities and that through routine clinical service. This potential bias is likely to be as a result of generalized poor socioeconomic levels, poor access to health care and low educational levels. This observation is confirmed by the fact that potential participants get disappointed if they are not included in the research activities. Studies in other research areas have documented similar findings [31-33]. It is likely that the perceived benefits will change over time with improving socio-economic levels or access to optimal health care. Discussions on KHRC research agenda were mainly requests to do further research into other diseases of importance to community members based on their perception of the disease severity or frequency. Community members also emphasized the need to ensure gender equity in health research since most KHRC research were usually conducted among women and children. KHRC is currently conducting baseline studies in hypertension and diabetes as a platform for further research in response to community needs.

Our aim was to engage the entire community members and their leaders as the “community” for the CE process. We therefore used various methods such as radio discussions, FGDs and individual discussion in the CE process. It was challenging to estimate the proportion of the population that listened to the radio discussions though a number of calls came in during the programme to discuss KHRC’s research agenda. The use of radio discussion were limited to those who could afford to call into the radio discussions, and probably of a relatively higher socioeconomic class. We however, engaged other social minority groups such as women with young children and adolescents in focus group discussions. We believe that it would have been most ideal to engage the community in several focus group discussions or community meetings as this process may enhance the depth of discussions. To overcome the potentially expensive approach of engaging diverse groups of the community, establishing a community advisory group that will represent the community in CE activities with research centres may be appropriate.

## Conclusion

CE is an integral part of health research. KHRC has developed a culturally appropriate CE model based on mutual understanding with community members. The experience obtained in the CE process has contributed to building CE capacity among scientific staff and community members. Other health research institutions in developing countries could consider the experiences gained.

## Competing interests

All authors declared that they have no competing interest.

## Authors’ contributions

KPA conceived the idea. KPA, CTA and SOA wrote the proposal. All authors contributed to the study’s implementation. KPA, CTA and CKD wrote the first draft of the paper and the final version was reviewed and approved by all authors. SOA who is also the Director of the Kintampo Health Research Centre approved for the paper to be submitted.

## Pre-publication history

The pre-publication history for this paper can be accessed here:

http://www.biomedcentral.com/1472-6963/13/383/prepub
